# A Rare Case of Persistent Bacteremia: Leadless Micra Pacemaker Endocarditis

**DOI:** 10.1155/2023/8326020

**Published:** 2023-01-18

**Authors:** Himax Patel, Sean Harrell, Haitham Hreibe, Musa Sharkawi, Wael AlJaroudi

**Affiliations:** Augusta University Medical Center, 1120 15th Street, Augusta, GA 30912, USA

## Abstract

Leadless pacing systems have revolutionized the field of electrophysiology given its low complication rates and almost non-existent rate of infections compared with traditional pacemakers. These devices boast resistance to infections given its unique features; however, as described in this report, device-related infection from these leadless devices is still possible. In patients with leadless pacing system that is persistently bacteremic in the future, evaluation of the device with transesophageal echocardiogram or intracardiac echocardiography should be performed, and if vegetation is noted on the device, device extraction should highly be considered, along with empiric intravenous antibiotics. Lastly, new leadless device should not be re-implanted within 2 weeks of the removal of the infected device to prevent seeding of the new device.

## 1. Introduction

Every year millions of pacemakers are implanted worldwide, and with our aging population, this number is expected to rise significantly over the next decade. Leadless pacing systems have revolutionized the field of electrophysiology given its miniature size and significantly low complication rates compared with traditional pacemakers. In addition, infections with leadless pacing systems are almost non-existent. Therefore, these devices are an attractive and safe pacing alternative to traditional pacemakers for clinicians around the world. In this case report, we report an exceptionally rare leadless (Micra, Medtronic, Minneapolis, Minnesota) pacemaker endocarditis with successful removal of the device three months after its implantation.

## 2. Case Report

A 66-year-old male was originally admitted to the cardiovascular intensive care unit (CVICU) for the management of undifferentiated shock. His medical history was significant for hypertension, hyperlipidemia, chronic obstructive pulmonary disease, polysubstance abuse of methamphetamine and tobacco, and atrial flutter status post-ablation. Patient was found unresponsive in his car near a gas station prior to the emergency medical services bringing him in the hospital. Upon arrival to the emergency department (ED), he was noted to be hypotensive and hypoxic. He was promptly intubated, started on vasopressors, and broad-spectrum antibiotics [intravenous route (IV) vancomycin 1000 mg every eight hours and IV cefepime 2 g every eight hours] for suspicion of septic shock were initiated. His physical exam was significant for regular rate and rhythm, coarse breath sounds, and cool extremities. His labs revealed a white blood count of 19,200 mm^3^, creatinine 2.18 mg/dL, aspartate transaminase 530 U/L, alanine transaminase 396 U/L, lactic acid 6.2 mmol/L, and troponin I 0.29 ng/mL. Urine drug screen was positive for methamphetamines. Electrocardiogram noted normal sinus rate and rhythm with old right bundle branch block. While in the ED, patient had multiple episodes of non-sustained ventricular tachycardia and intermittent episodes of supraventricular tachycardia, stabilized with direct current cardioversion and amiodarone infusion. Upon arrival to the CVICU, a transthoracic echocardiogram (TTE) was performed, which noted a reduced left ventricular ejection fraction (LVEF) of 35–40%, severely dilated right ventricle (RV), and reduced RV function with RV S' velocity 6.7 cm/second and tricuspid annular plane systolic excursion 1.4 cm, with bowing of the RV septum and dilated inferior vena cava. His troponin I continued to uptrend to 39 ng/mL. Given all these findings, an additional component of cardiogenic shock secondary to type I non-ST elevation myocardial infarction (NSTEMI), in the setting of amphetamine abuse, was suspected. CVICU course complicated by episodes of hemodynamic unstable junctional bradycardia with rate of 30 seconds. In the context of cardiogenic shock, type I NSTEMI, and hemodynamic unstable bradycardia, patient underwent left heart catheterization, along with placement of temporary venous pacing wires. Coronary angiogram revealed severe left anterior descending artery and right coronary artery disease with TIMI 3 flow throughout the coronary vessels suitable for medical management. Course was further complicated by episodes of atrial fibrillation with rapid ventricular rates in 140–150 seconds. These episodes were managed with amiodarone boluses. Given episodes of occasional supraventricular tachycardia, with intermittent episodes of bradycardia, patient ultimately underwent placement of leadless RV pacemaker (Micra, Medtronic) for management of his tachycardia–bradycardia syndrome. After placement of the leadless pacemaker, patients' atrial fibrillation was controlled with oral amiodarone without complications. Upon clinical stability, patient was transferred to medicine service for further management.

**Figure 1:**
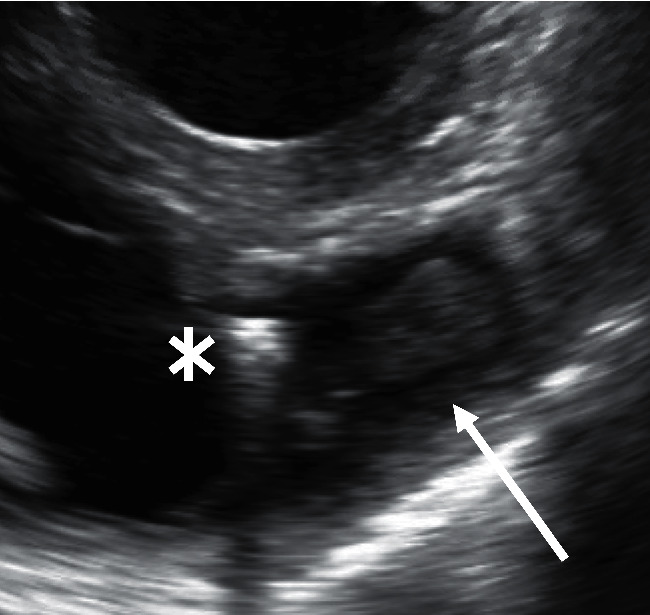
Transesophageal echocardiography image of Micra device (asterisk) with attached vegetation (arrow).

**Figure 2:**
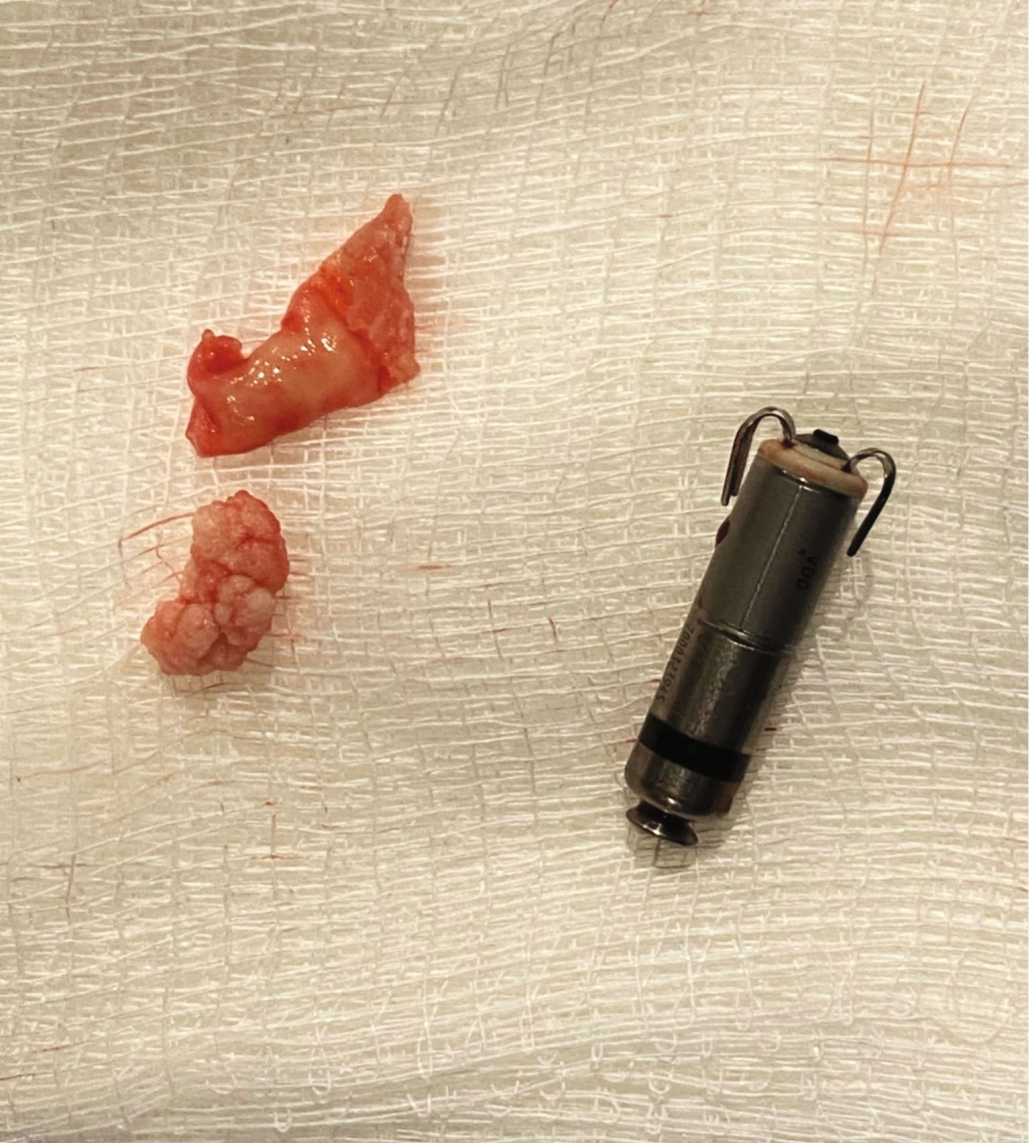
Gross image of successfully removed Micra device and previously attached vegetation.

While awaiting rehabilitation placement on the medicine service, forty-nine days post Micra placement, patient developed a new fever; however, his white blood cell count was noted to be normal ranging 4.4–7.3 thousand/mm^3^. Four sets of blood cultures (two aerobic bottles and two anaerobic bottles) were collected, and patient was found to have methicillin resistant *Staphylococcus aureus* (MRSA) bacteremia. Repeat cultures were collected forty-eight hours apart, with patient remaining bacteremic for seven days with clearance noted on the eighth day. He was initially treated with intravenous vancomycin 1000 mg every eight hours; however, he persistently remained febrile and bacteremic; therefore, after six days on vancomycin, his antibiotic regimen was escalated to IV daptomycin 680 mg daily and IV ceftaroline 600 mg every twelve hours for a total of six-week duration of therapy (see Table 1 for antibiotic susceptibility). Endocarditis was suspected, and patient underwent TTE, which noted a recovered LVEF of 50–55%, with thickened mitral leaflets, with no obvious vegetation. However, given high suspicion for endocarditis, thickened mitral valves, and persistent bacteremia, patient underwent transesophageal echocardiography (TEE), which noted a mobile mass, measuring 1.6 cm × 1.8 cm attached to the leadless Micra pacemaker seen near the right ventricular outflow tract part of the RV under the pulmonary valve (Figure 1). No other vegetations were noted. Given this finding, patient underwent debulking of the vegetation along with removal of the infected Micra leadless pacemaker. This procedure was performed by the electrophysiology team in conjunction with interventional cardiology. Bilateral venous access was obtained, and a 26-Fr × 33-cm sheath was placed into the right common femoral vein. Access from the left common femoral vein was used to place an intracardiac echo catheter to direct the device. Subsequently, 7-Fr balloon-tipped catheter was placed into the left pulmonary artery. A stiff Amplatz wire was placed in the distal left pulmonary artery branch, over which a T-24 FlowTriever aspiration catheter was advanced. Aspirations in the pulmonary artery, RV outflow tract, as well as the RV were performed, for a total of eight aspirations. T-20 deflectable catheter was also used to perform further aspiration of vegetation and extirpation of matter from the locations mentioned earlier. After majority of debulking was performed, a J-wire was advanced in the pulmonary artery, and over the wire, a FlexCath (Medtronic) guiding wire was advanced into the right atrium and then into the RV. The FlexCath was directed towards the leadless pacemaker, and a 25 mm gooseneck snare was advanced to the leadless pacemaker and the proximal groove on the leadless pacemaker was then snared and the device was aligned with the guiding catheter in an axial position. Using gentle and sustained traction, the leadless pacemaker catheter was removed from the heart and kept snared until it was retrieved into the sheath. At that point, the sheath with the leadless pacemaker in it was removed, and hemostasis was achieved with manual pressure. At the end of the procedure, intracardiac echo assessment revealed no pericardial effusion with no residual endocarditis. The Micra device and associated vegetation were removed and placed next to one another (Figure 2). The catheters and sheaths were removed without issue. After removal of the vegetation and the infected device, patient's blood cultures remained negative. He was treated for six weeks of antibiotics (daptomycin and ceftaroline) with rest of patient's hospital course remaining unremarkable. The patient currently awaits placement at a subacute rehabilitation facility.

## 3. Discussion

Cardiac pacemaker technology has advanced tremendously since its first introduction in the 1950s. Traditional pacemakers with transvenous leads are still very commonly used; however, in patients only requiring single chamber pacing, more clinicians are leaning towards using the newer leadless pacing devices given their significantly lower rate of complications and infections compared with traditional devices. To date, there are two Food and Drug Administration approved leadless pacing systems available, Micra pacing system by Medtronic, and Avier VR by Abbott. Micra was approved in 2016, with Avier VR getting approval for use in April 2022. They both allow for RV pacing, sensing, and rate responsiveness. The leadless pacing system works similar to traditional pacemakers with added advantage of it being leadless and not needing a pacemaker pocket. Avoiding lead components and pacemaker pocket can prevent many common complications that are classically associated with traditional pacemakers, such as tricuspid regurgitation, lead fractures, pocket site infections, and most importantly pacemaker endocarditis. Reported incidence rate of traditional pacemaker system-related infection is between 0.3 and 12.6% [1]. The new leadless pacemakers boasts no cases of infection reported in clinical trials enrolling over 3000 patients [2].

There are many factors that make the leadless pacing system resistant to infections. Leadless pacing devices have 4–5× smaller surface area in the bloodstream than traditional pacemakers, which allows for a lesser area for bacterial adhesion; in addition, absence of the pocket and leads further reduces bacterial adhesion sites. The Micra leadless pacemaker system is made of a titanium, which is coated by a layer of parylene. In a recent study by El-Chami et al., authors noted that parylene coating of devices significantly reduced bacterial growth of *S. aureus* and *Pseudomonas aeruginosa*, which further explains low infection rates with these devices [3]. Lastly, the leadless pacing system is deployed in the RV, where it is surrounded by the turbulent flow of the RV. The high turbulence prevents blood stagnation; therefore, not allowing bacteria any opportunity to adhere to the device. Through these mechanisms, the low rate of leadless pacemaker infections can be explained.

In a recent meta-analysis by Ngo et al., authors noted that leadless pacemakers, especially Micra was associated with low incidence of complications at ninety days (0.46%) and one year (1.77%) after implantation [4]. When individual complications with leadless devices were considered, in a pooled data of 12 studies with 2376 patients, only 1 report of “sepsis” was reported after implantation of the leadless pacing system, which was successfully treated with intravenous antibiotics [4, 5]. No other reports of infection related to the device implantation were reported. In addition to its safety profile, studies have also reported an improvement in quality of life, physical activity, and mental health with leadless pacing systems compared with traditional devices [6, 7].

To the best of our knowledge, there are only two other case reports published in the literature related to leadless pacing system endocarditis. In the case of Ellison et al., a 37-year-old female underwent placement of leadless Micra pacing system for neurocardiogenic syncope. A month post-implantation patient was noted to have fevers of unknown etiology. During extensive workup, leadless pacemaker was found to be the cause of her infection after they noted 1.3 cm × 0.5 cm frond like material on the device. Patient underwent extraction of the device with resolution of the infection [8]. The second report by Koay et al. describes an 80-year-old who underwent placement of leadless Micra system for tachycardia–bradycardia syndrome. A month later, patient presented to the hospital with sepsis secondary to MRSA bacteremia which was treated with intravenous antibiotics. TEE was significant for a vegetation measuring 1.2 cm × 0.9 cm attached to the proximal site of the device. Patient remained persistently bacteremic on antibiotics; therefore, patient ultimately underwent device extraction. Post-extraction, patient completed antibiotic therapy with resolution of her bacteremia [9].

Even though rare, leadless pacing system infection is possible. If device-related infection is noted, removal of the infected device is recommended. Both Micra and Avier VR are designed with a proximal retrieval feature to assist during the removal of the device. In regard to the re-implantation of a new device after removal of an old, infected device, guidance can be obtained from the guidelines for the infected traditional pacemaker systems. Based on those guidelines, new leadless device should not be re-implanted within 2 weeks of the removal of the infected device to prevent further complications [10].

## 4. Conclusions

Leadless pacing systems have revolutionized the field of electrophysiology since its first introduction in 2016 given its low complication rates and almost non-existent rate of infections compared with traditional pacemakers. These devices are resistant to infections given their smaller surface area, no need for pocket site and transvenous leads, parylene coating, and lastly their location in the highly turbulent RV. However, as described in this report, device-related infection from these leadless devices is still possible. In patients with leadless pacing system that are noted to be persistently bacteremic in the future, evaluation of the device with transesophageal echocardiogram or intracardiac echocardiography should be considered. If vegetation is noted on the device, device extraction should highly be considered, along with empiric intravenous antibiotics. Lastly, new leadless device should not be re-implanted within 2 weeks of the removal of the infected device to prevent seeding of the new device.

## 5. Teaching Points

1. Leadless pacing system has significantly lower complication rates, especially infections, compared with traditional devices.
2. Infections with leadless pacemakers are extremely rare; however, can still occur.
3. If leadless device-related infection or vegetation is noted, device extraction should be considered, in addition to intravenous antibiotic therapy.
4. New leadless pacing system should be re-implanted no sooner than 2 weeks after removal of the infected device.

## Figures and Tables

**Table 1:** MRSA susceptibility report.

Drug	Susceptible/resistant
Clindamycin	Susceptible
Daptomycin	Susceptible
Doxycycline	Susceptible
Erythromycin	Resistant
Gentamycin	Susceptible
Oxacillin	Resistant
Rifampin	Susceptible
Trimethoprim/sulfamethoxazole	Susceptible
Vancomycin	Susceptible

## Data Availability

Data supporting this research article are available from the corresponding author or first author on reasonable request.
